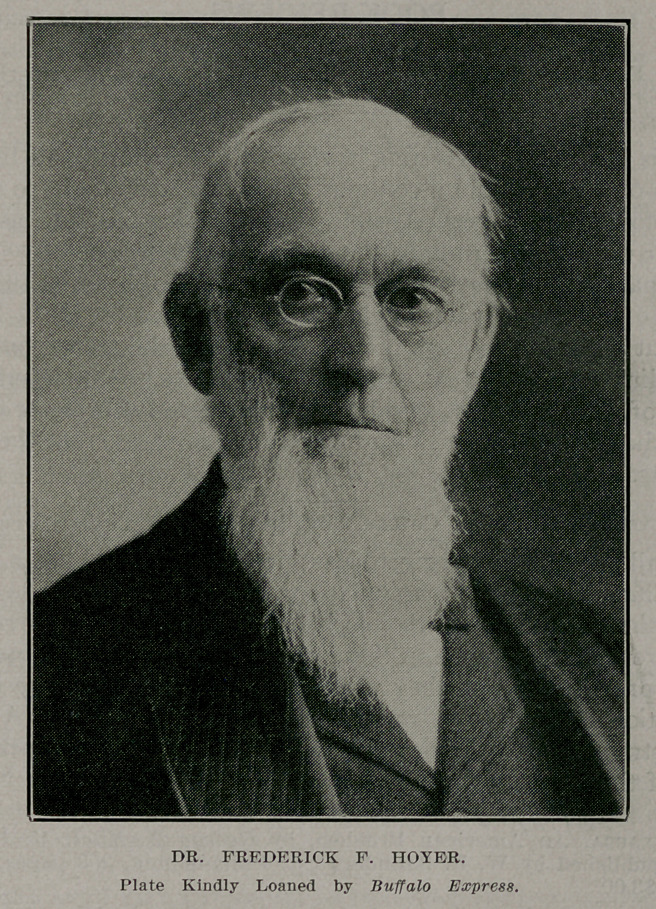# Dr. Frederick F. Hoyer

**Published:** 1912-09

**Authors:** 


Dr. Frederick F. Hoyer of Tonawanda, a graduate of the University of Buffalo, 1849, died Aug. 16, 1912, aged 91.

Dr. Hoyer's great, great grandfather was Peter Hoyer, who migrated from Hesse Cassel in 1748 and located in Herkimer. Dr. Hoyer was born at the family home in Warren (originally known as Andreastown), Herkimer Co., N. Y., May 9, 1822. After a course of medicine at the old Geneva Medical College, he practiced by license, in accordance with the existing custom and law, with his uncle, Dr. Peter P. Murphy of Roylaton. In October, 1846, he married Miss Pauline Towne of Royalton who died in 1910, aged 87. After marriage he continued his medical studies and received the doctor's degree at the University of Buffalo in 1849. On May 9, 1849, he and his wife moved to Tonawanda by canal boat, built their home there in 1853, and resided there till their death. From 1861 to 1863, he was visiting physician to the Erie Co. Almshouse. In 1880 he was President of the

Medical Society of the County of Erie. In 1908, he was made an honorary member. Dr. Hoyer was the oldest mason in the state, having joined Lake Erie Commandery (of which his uncle was one of the three founders) in 1857. For several years, his masonic brethren honored him by calling in a body at his home. Dr. Hoyer, beside enduring the hardships of pioneer practice, common to his time, had the experience of two epidemics of cholera Asiatica and of numerous conflicts with small pox. He

DR. FREDERICK F. HOYER.

Plate Kindly Loaned by *Buffalo Express.*

was a strong man, physically, mentally and spiritually; rightly honored and respected by his patients, to the third and fourth generation, by his profession, by his fellow citizens.

## Figures and Tables

**Figure f1:**